# Postoperative subdural hematoma as a rare complication of non-traumatic craniotomy: Two cases report

**DOI:** 10.1097/MD.0000000000023589

**Published:** 2021-02-12

**Authors:** Xianfeng Gao, Huibo Liu, Wanzhen Xu, Yang Sun, Yang Zhang, Xiaobo Zhu, Wei Wang

**Affiliations:** a Department of Neurosurgery; b Department of Dermatology, First Hospital, Jilin University, Changchun, China.

**Keywords:** non-traumatic craniotomy, postoperative intracranial hemorrhage, postoperative subdural hematoma

## Abstract

**Rationale:**

Postoperative intracranial hemorrhage is a serious and even fatal complication after non-traumatic craniotomy, in which epidural hematoma and intracerebral hematoma are relatively common. Postoperative subdural hematoma is rare, and its pathogenic mechanism remains unclear.

**Patient concerns:**

In the present study, we report 2 cases with postoperative subdural hematoma after non-traumatic craniotomy.

**Diagnoses:**

The diagnosis of acute subdural hematoma (aSDH) was rendered according to the imaging features.

**Interventions:**

Hematoma evacuation was performed immediately.

**Outcomes:**

Two months later, the first patient continued to have impaired consciousness and sensorimotor deficiency in the right extremities. And the second one remained unconscious and continued to have sensorimotor disturbance in the right extremities after 6 weeks of rehabilitation.

**Lessons:**

Neurosurgeons should be aware of potential subdural hematoma after non-traumatic craniotomy, since this condition is usually latent and associated with poor prognosis. Early identification and surgical evacuation should be highlighted.

## 1 Introduction

Postoperative intracranial hemorrhage is a serious and even fatal complication after non-traumatic craniotomy, in which epidural hematoma and intracerebral hematoma are relatively common. Postoperative subdural hematoma is rare, and its pathogenic mechanism remains unclear. This condition is usually latent. Hence, early diagnosis remains challenging.

Previously published literatures in PubMed were reviewed using the following search terms: “subdural hematoma AND postoperative AND non-traumatic craniotomy.” No article specially describing postoperative subdural hematoma was retrieved. Palmer et al^[1]^ reviewed 6668 patients who underwent craniotomy and found that 71 patients developed postoperative intracranial hemorrhage yielding a hemorrhage rate of 1.1%. Furthermore, patients with epidural hematoma or intracerebral hematoma accounted for 76% of all hemorrhage cases, while patients with subdural hematoma only accounted for 5% of these cases. Kalfas et al^[2]^ reviewed 4992 patients who underwent craniotomy, and 40 patients developed postoperative intracranial hemorrhage, in which subdural hematoma only accounted for 7.5%.

In the present study, we report 2 cases with postoperative subdural hematoma after non-traumatic craniotomy and discussed the potential risk factors.

## 2 Case report

### 2.1 Case 1

**Figure 1:**
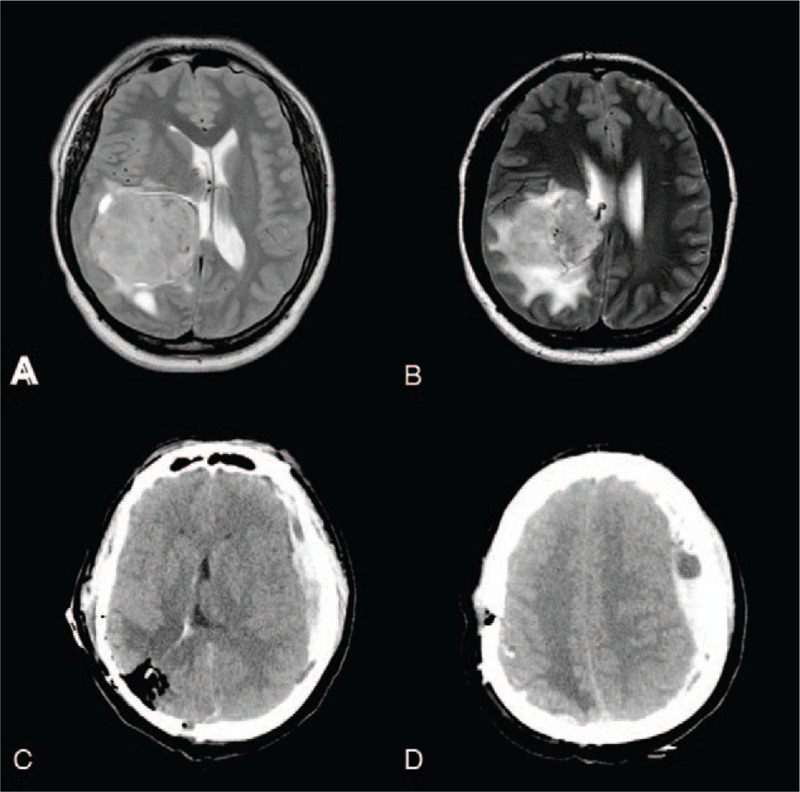
Radiological profiles of Case 1. (A–B) Axial T2-weighted MRI revealed a lesion in the right posterior horn of the lateral ventricle, with remarkable peritumoral edema. (C–D) Postoperative CT revealed a contralateral subdural hematoma. CT = computed tomography, MRI = magnetic resource imaging.

A 35-year-old man presented with a 3-year history of headache. Magnetic resonance imaging (MRI) revealed a 5.5 × 6.0 × 5.3 cm lesion in the posterior horn of the lateral ventricle, with remarkable peritumoral edema (Fig. 1A and B). Physical and laboratory examinations on admission were all normal. The previous medical history was unremarkable, except for transient meningitis during the patient's childhood. A microscopical tumorectomy was performed via occipital approach in a lateral decubitus position. Intraoperatively, the hemostasis was performed carefully, and the dura mater was tightly sutured. Blood pressure and heart rate were all normal throughout the surgical procedures. However, during anesthetic resuscitation, mydriasis was noted in the right eye. Postoperative hemorrhage was suspected. Immediate computed tomography (CT) revealed a contralateral subdural hematoma (Fig. 1C and D). A second craniotomy was performed for evacuation of hematoma. Then, a bridging vein in the temporoparietal region was intraoperatively found to be ruptured with active bleeding. The bone flap was removed for decompression. Two months after the operation, the patient continued to have impaired consciousness and sensorimotor deficiency in the right extremities.

### 2.2 Case 2

**Figure 2:**
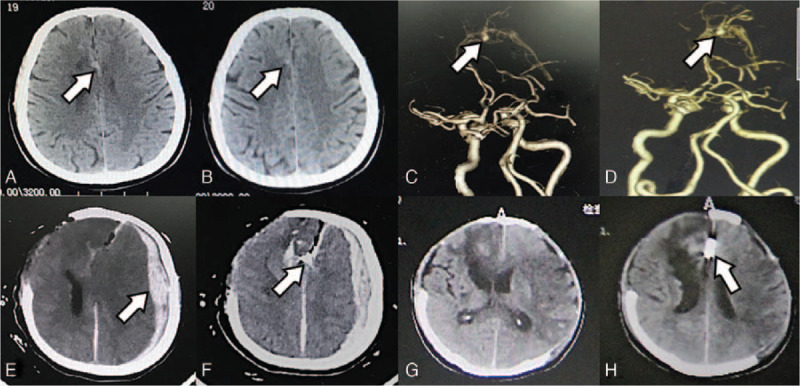
Radiological profiles of Case 2. (A–B) CT revealed hyperintensity involving the right cerebral falx. (C–D) CTA revealed an aneurysm in the distal anterior cerebral artery. (E–F) Postoperative CT revealed an acute contralateral subdural hematoma with a significant midline shift. (G) CT after the second operation and (H) repeated CT after 2 weeks revealed that the hematoma was completely evacuated. CT = computed tomography, CTA = CT angiography.

A 52-year-old man presented with a 10-day history of headache and dizziness. Computed tomography (CT) revealed hyperintensity involving the right cerebral falx (Fig. 2A and B). Computed tomographic angiography (CTA) revealed an aneurysm in the distal anterior cerebral artery (Fig. 2C and D). There was no history of trauma, hypertension, or hemorrhagic disease. Physical and laboratory examinations on admission were all normal. Microscopical aneurysm clipping was performed via the anterior interhemispheric fissure approach in the lateral decubitus position. During bone flap opening, the tension of the dura mater was high, and blood pressure and heart rate were fluctuating. Aneurysm rupture and bleeding were suspected. However, after the bone flap was completely opened, the tension of the dura mater decreased, and the cerebral tissue was swollen without encephalocele. The aneurysm was successfully clipped, and the bone flap was removed. Postoperatively, mydriasis was noted in the contralateral eye. Emergent CT revealed an acute contralateral subdural hematoma with a significant midline shift (Fig. 2E and F). A second craniotomy was performed for evacuation of hematoma. However, intraoperatively, the operators failed to identify the bleeding vessel. The postoperative CT scan revealed that the hematoma was completely evacuated (Fig. 2G and H). After 6 weeks of rehabilitation, the patient remained unconscious and had sensorimotor disturbance in the right extremities.

Our study protocol was approved by the Ethics Committee of the First Hospital of Jilin University (No. 2017-440), and the informed written consent was obtained from the patient for publication of this case report and accompanying images.

## 3 Discussion

In the present study, 2 patients developed contralateral subdural hematoma after non-traumatic craniotomy. This condition is extremely rare, with an estimated incidence of 55 to 60‰, according to literature.^[1,2]^ The definitive pathogenesis of remote hemorrhage following craniotomy remains to be elucidated. The mainstream views have considered that there may be 4 risk factors: intraoperative hemodynamic changes, especially the fluctuation of blood pressure; preoperative coagulation disorders or abnormal hepatic functions; intraoperative excessive loss of cerebrospinal fluid; severe traction of brain tissues.^[3–6]^ It is noteworthy that the cerebral exposure and excessive loss of cerebrospinal fluid can lead to unbalanced intracranial pressure, thereby resulting in the mechanical shift of brain parenchyma, which may cause rupture in bridging veins or small reflux veins.^[7–10]^ These bridging veins are characterized by thin walls due to the lack of smooth muscle cells and collagenous fibers, increasing the risk of subdural hematoma.^[11]^ In addition, potential coagulation disturbances may also increase hemorrhage risk.

In Case 1, intraoperative findings revealed that a bridging vein in the contralateral temporoparietal region was ruptured with active bleeding, and there was no significant injury in the surface of the local cerebral tissue. Generally, craniotomy does not cause traction of the contralateral vessels, especially via the occipital approach. The bridging vein connects the brain surface and dural sinuses, which has a certain toughness and flexible space, and only a severe cerebral contusion/laceration or shift can lead to rupture of the bridging vein.^[11]^ In addition, the patient had a previous history of meningitis during childhood, which may also increase the risk of bridging vein rupture. Inflammation can increase the fragility of blood vessels and local tissue adhesions, and infection can directly or indirectly induce vascular sclerosis.^[12–14]^ During the primary tumorectomy operation, vascular sclerosis and tissue adhesions were also noted, which is unusual in young patients. Moreover, the removal of the tumor reduced the cerebral volume, and the secondary decrease in intracranial pressure might lead to brain shift, eventually resulting to bridging vein rupture.^[15–18]^ However, the above hypothesis of mechanical traction is more likely to be associated with epidural hematoma rather than subdural hematoma. Thus, the investigators considered that the previous history of meningitis may be a crucial risk factor.^[14]^

In Case 2, the contralateral subdural hematoma was limited, and there was no cerebral injury, suggesting that the hemorrhage had mild or moderate impact to brain parenchyma. These features were consistent with exogenous or venous hemorrhage. During the aneurysm clipping operation, a temporary intracranial hypertension was noted, which was considered to be caused by aneurysm rupture. The unbalanced intracranial pressure and gravity may lead to the aggregation of blood in the contralateral subdural space, forming exogenous hematoma. In literature, subdural hematoma has been frequently observed in patients with ruptured aneurysm in the A2 segment, and this also supports the present hypothesis.^[19,20]^ Furthermore, during the hematoma evacuation, bridging veins refluxing to the sagittal sinus were all normal. Hence, venous hemorrhage was excluded.

In brief, it was considered that the subdural hematoma in the 1st case was due to inflammatory changes related to bridging vein rupture, and the hematoma in the 2nd case was caused by aneurysm rupture and ectopic blood aggregation. Subdural hematomas following non-traumatic craniotomy are severe.^[21]^ Preoperative comprehensive evaluations should be highlighted, especially coagulation function and previous history of craniocerebral diseases. Intraoperatively and postoperatively, neurosurgeons should be vigilant against intracranial hypertension and prevent excessive loss of cerebrospinal fluid. In addition, except for mechanical traction factors, several underlying diseases may also increase the risk of postoperative hemorrhage, such as hypertension, coagulation disturbance, cirrhosis, scleratheroma, and vascular malformations.^[1,2]^ Since the prognosis of postoperative remote hematoma is usually unfavorable, early diagnosis and timely surgical treatment should be emphasized. In other words, the neurosurgeons should be aware of the potential subdural hematoma after non-traumatic craniotomy, since this condition is usually latent and associated with unfavorable prognosis. Early diagnosis and timely surgical evacuation should be highlighted.

## Author contributions

XXXX.

## Abbreviations

Abbreviations: aSDH = acute subdural hematoma, CT = computed tomography, CTA = CT angiography, DSA = digital subtraction angiography, MRA = magnetic resource angiography, MRI = magnetic resource imaging.
